# Fluid management guided by a continuous non-invasive arterial pressure device is associated with decreased postoperative morbidity after total knee and hip replacement

**DOI:** 10.1186/s12871-015-0131-8

**Published:** 2015-10-15

**Authors:** Jan Benes, Lenka Haidingerova, Jiri Pouska, Jan Stepanik, Alena Stenglova, Jan Zatloukal, Richard Pradl, Ivan Chytra, Eduard Kasal

**Affiliations:** Department of Anesthesia and Intensive Care Medicine, Teaching Hospital and Faculty of Medicine in Plzen, Charles University Prague, alej Svobody 80, 306 40 Plzen, Czech Republic

**Keywords:** Fluid management, Perioperative care, Goal directed therapy, Pulse pressure variation

## Abstract

**Background:**

The use of goal directed fluid protocols in intermediate risk patients undergoing hip or knee replacement was studied in few trials using invasive monitoring. For this reason we have implemented two different fluid management protocols, both based on a novel totally non-invasive arterial pressure monitoring device and compared them to the standard (no-protocol) treatment applied before the transition in our academic institution.

**Methods:**

Three treatment groups were compared in this prospective study: the observational (CONTROL, *N* = 40) group before adoption of fluid protocols and two randomized groups after the transition to protocol fluid management with the use of the continuous non-invasive blood pressure monitoring (CNAP®) device. In the PRESSURE group (*N* = 40) standard variables were used for restrictive fluid therapy. Goal directed fluid therapy using pulse pressure variation was used in the GDFT arm (*N* = 40). The influence on the rate of postoperative complications, on the hospital length of stay and other parameters was assessed.

**Results:**

Both protocols were associated with decreased fluid administration and maintained hemodynamic stability. Reduced rate of postoperative infection and organ complications (22 (55 %) vs. 33 (83 %) patients; *p* = 0.016; relative risk 0.67 (0.49–0.91)) was observed in the GDFT group compared to CONTROL. Lower number of patients receiving transfusion (4 (10 %) in GDFT vs. 17 (43 %) in CONTROL; *p* = 0.005) might contribute to this observation. No significant differences were observed in other end-points.

**Conclusion:**

In our study, the use of the fluid protocol based on pulse pressure variation assessed using continuous non-invasive arterial pressure measurement seems to be associated with a reduction in postoperative complications and transfusion needs as compared to standard no-protocol treatment.

**Trial registration:**

ACTRN12612001014842

**Electronic supplementary material:**

The online version of this article (doi:10.1186/s12871-015-0131-8) contains supplementary material, which is available to authorized users.

## Background

In recent years fluid management protocols and goal-directed therapy (GDT) have gained popularity among anesthesiologists [1]. According to recent meta-analyses GDT is associated with a decreased rate of postoperative complications [2–4] and fluid optimization protocols seem to be the necessary step [5]. A rational fluid optimization protocol consisting of maintenance infusion covering basal loss and goal-directed top-ups has been proposed by other authors [6, 7]. However, according to a recently published survey among European and American anesthesiologists [8], protocols for perioperative hemodynamic care are lacking in many institutions. Another important factor stressed by this survey was the use of dynamic variations of stroke volume or its surrogates as substitutes of cardiac output monitoring. In high-risk surgical populations, the studies by Forget [9] and Lopes [10] as well as one large meta-analysis of other published trials [11] showed a positive influence of the dynamic variations-led GDT even without monitoring of cardiac output. The adoption of dynamic variations is limited in some situations [12], but can significantly simplify the delivery of fluid optimization protocols.

Total hip or knee replacements are usually performed among elderly patients, but neither the chronic health state nor the procedure-associated risk usually exceed moderate risk. In one study, hemodynamic optimization using oxygen delivery targets was associated with favorable postoperative outcome [13]. However, such invasive monitoring might lead to some minor complications [14], increases the economic burden and is often deemed unnecessary. The volume clamp method for continuous non-invasive arterial pressure monitoring or similar techniques enables the delivery of a fluid therapy protocol without these disadvantages.

In this trial we studied the effect of a controlled transition from non-protocolled to protocolled fluid management on the rate of postoperative complications and other relevant perioperative outcomes in patients undergoing elective total hip and knee replacement. Our hypothesis was that the fluid management protocol guided by non-invasive continuous arterial pressure and the easily obtained dynamic predictor of fluid responsiveness (respiratory variation of the pulse pressure - PPV) would help to decrease postoperative morbidity.

## Methods

The study was performed at the Department of Anesthesia and Intensive Care Medicine of the Faculty of Medicine and Charles University Hospital in Plzen. The Institutional review board approved the study which was consequently registered in the primary WHO register (ACTRN12612001014842), and all patients gave and signed the informed consent. The study was performed and reported in accordance with the CONSORT statement [15] (see Additional file 1). The trial followed a two-stage “before and after” design with one control observational group and two randomized-protocol based study groups. All groups consisted of two strata (total hip replacement - 20 patients and total knee replacement – 20 patients). Basic inclusion for all study participants were: age above 18, general anesthesia, regular heart rhythm, informed consent and no need for direct and continuous blood pressure monitoring or advanced hemodynamic monitoring.

### First stage – observational and wash-out period

The CONTROL group was treated according to the usual care without any fluid protocol. On the day of surgery patients were fasted and received 2 ml/kg/h of crystalloid infusion from the morning until transport to the operation room. Throughout the surgery monitoring of blood pressure was performed using an automatic oscillometric non-invasive arm cuff (rate of measurement once every 5 min but there were no restrictions in increasing the rate). Blood pressure fluctuations within 20 % of the baseline values were tolerated. The amounts of fluids infused were at the discretion of the treating anesthesiologist. Transfusion of red blood cells was indicated when the hemoglobin fell below 90 g/l; in overall healthy patients lower thresholds (up to 70 g/l) were tolerated. The blood loss was assessed by measuring the balance of closed system suction. Two independent investigators (JZ and RP) were responsible for the assessment of the CONTROL group patients. These two investigators did not participate in the following course of the study so the outcome data was concealed.

After completion of the first phase a wash-out period was interposed. Within this time all study members responsible for in-study anesthesia delivery (LH, JP and JS) were trained in the use of the CNAP® monitor and the protocol (each of them had to perform at least 10 cases per protocol group).

### Second stage – randomized, protocol based

During the second stage all patients undergoing scheduled total knee or hip replacement fulfilling mentioned inclusion criteria were found eligible. Patients were equally randomized into two groups (GDFT and PRESSURE) each with 40 patients, stratified to knee and hip replacement (20 patients each). Randomization was performed by the study member responsible for the anesthesia delivery before the induction using sealed opaque envelope technique stored in non-transparent containers (one per stratum) with group allocation in a 1:1 ratio. Each envelope, holding one patient’s identification, was then returned into another non-transparent container which remained sealed till the end of the study when the concealment was broken for statistical analysis. This made all other study members as well as the surgeon and other health care staff blinded to individual patient’s allocation.

### Anesthesia, monitoring and protocol delivery

All patients were fasted before the procedure, small amounts of liquids were allowed for those later on the operating schedule and for chronic medication ingestion. During fasting all patients received an infusion of Hartmann solution (2 ml/kg/h) from the morning of the operative day. General anesthesia was induced using propofol (2 mg/kg) and sufentanil (0.2 μg/kg) and tracheal intubation was facilitated by atracurium or rocuronium (0.5 mg/kg). Volatile anesthetic (sevoflurane – MAC 0.8–1.2 accounted for age) in oxygen-N_2_O mixture was used for anesthesia maintenance. Opioid or muscle relaxant increments were used to secure adequate analgesia and operating conditions. Under relevant circumstances a deviation in the usual induction or maintenance was tolerated at the discretion of the treating anesthesiologist or anesthesia consultant.

The CNAP® device (CNSystems, Graz, Austria) was used for blood pressure monitoring in both protocol groups (GDFT and PRESSURE). The device works utilizes the principle of volume clamping described by the Czech physiologist Peňáz in 1963 and adapted later by Fortin [16]. First, a state of vascular unloading is set by inflating the cuff around the finger to reach maximal pulse oscillations (the pressure inside and outside the arterial wall is then equivalent). Next, the volume of blood compartment of the finger is measured by plethysmography and held constant with the use of fast reacting inflations/deflations of the cuff. This enables a reconstruction of the arterial pressure curve at the level of the fingers. To obtain brachial pressure, the values are calibrated at the beginning and every 15 min thereafter using a standard non-invasive oscillometric measurement in the arm. The device displays blood pressure continuously, enabling the automatic calculation of the PPV (pulse pressure variation) and exports the pressure curve and values to standard monitor (Ultraview SL2700, Spacelabs Healthcare, Washington, USA). In the GDFT group all relevant data was shown to the anesthesiologist. In the PRESSURE group the screen of the CNAP® device was covered and the continuous arterial pressure curve and values (without PPV) were transferred to the bedside anesthesia monitor. The values of the PPV for the analysis were obtained off-line from stored data after the procedure by a study member (JB) blinded to patient allocation.

**Fig. 1:**
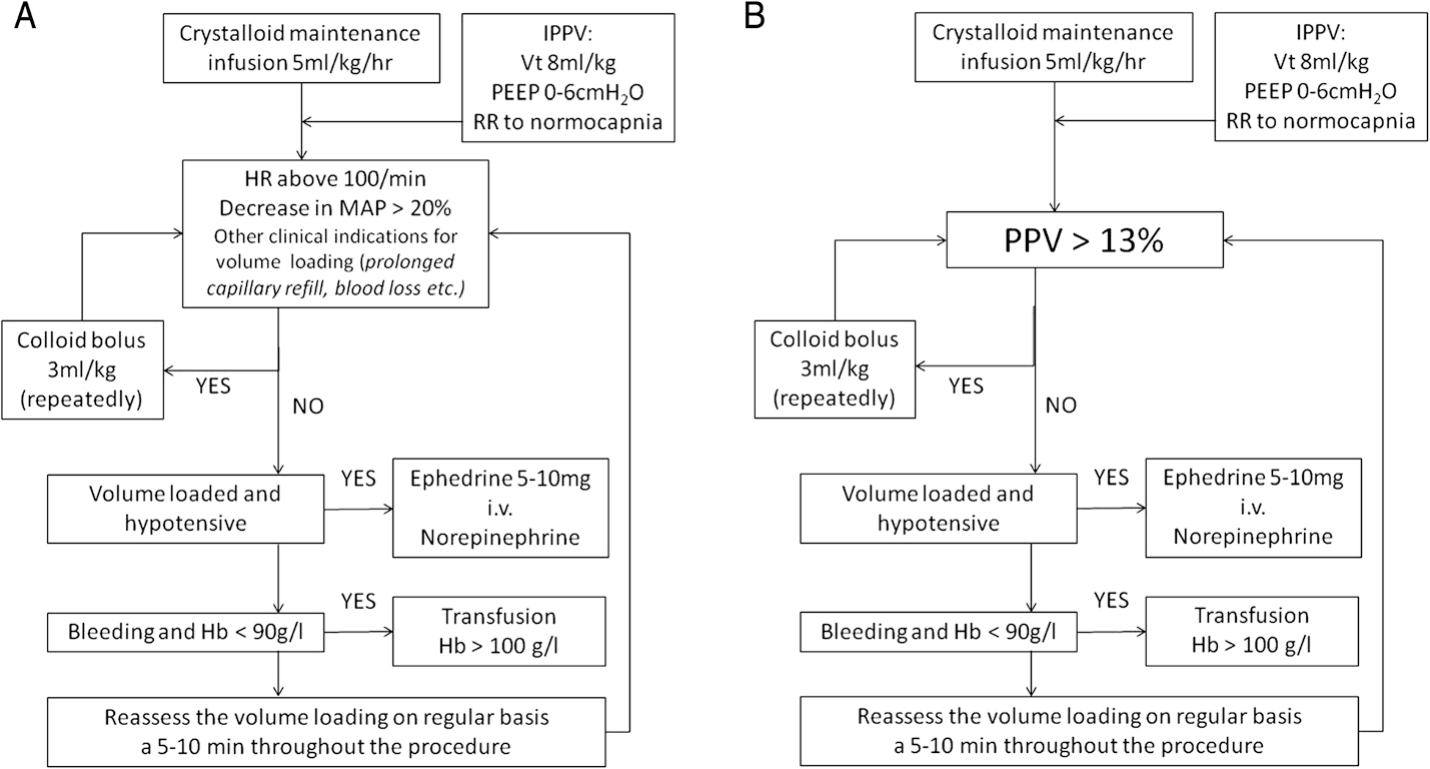
The protocols of fluid management in the second-stage groups (PRESSURE – panel **a**; GDFT – panel **b**). Legend: HR – hear rate; MAP – mean arterial pressure; PPV – pulse pressure variation; IPPV – Volume controlled ventilation; Vt – Tidal volume; PEEP – Positive End-Expiratory Pressure; RR – respiratory rate

Throughout the procedure fluid therapy was delivered according to the protocols displayed in Fig. 1a and b with maintenance infusion of 5 ml/kg/h of crystalloids (Plasmalyte, Baxter Czech s.r.o., Praha, Czech Republic). Repeated boluses of 3 ml/kg of colloids (preferentially Gelofusine 4 % or 130/0.4 HES 6 %, Volulyte; both B-Braun Melsungen, Germany) were used if indicated by usual pressure targets (PRESSURE group) or by pulse pressure variation above 13 % (GDFT group). In case of reaching the maximal dose of colloids (25 ml/kg) crystalloid boluses of 3 ml/kg would be used for further care. When the patient was hypotensive though reaching a “volume loaded state” (defined as PPV < 13 % or based on clinical assessment in the GDFT or PRESSURE groups respectively) a vasoactive rescue medication (Ephedrine 5–10 mg i.v. bolus or continuous Norepinephrine) was indicated. The decision which drug to choose was on the discretion of treating anesthesiologist based on presumed duration of hypotensive period and/or underlying cause. The number of hypotensive periods requiring volume loading and/or vasoactive treatment was collected as well as total dose of vasoactive medication. The same transfusion threshold (as the CONTROL group) was used if not required otherwise due to chronic conditions of the patient.

### Study outcomes

The number of patients with any postoperative organ or infectious complication was the primary outcome measure of the study. The list of relevant complications was based on previous GDT trials [13, 17] (Additional file 2). The treating physician was responsible for the diagnosis and treatment of all complications. Hospital length of stay and all-cause mortality were assessed as secondary outcomes. Fluid balance and lactate levels in the early (24 h) postoperative period were regarded as safety measures. Besides the mentioned conditions, other potentially relevant outcomes (i.e. ICU length of stay, duration of ventilator support, number of blood products used, hemoglobin level and hemodynamic profile in the intraoperative and early postoperative period as well as vasoactive medication used) were assessed. As both the ICU and hospital lengths of stay might be influenced by many other factors not relevant to the health care conditions of the patient, a readiness for standard ward transfer and for hospital discharge were also evaluated (screening criteria are listed in the Additional file 2). Two investigators (JB, AS), blinded to study group allocation and not participating in the anesthesia care and randomization, evaluated the state of the patients during regular visits.

### Statistical analysis

The number of CONTROL group patients was based on previous observations in similar patient population [13]. A significant reduction of postoperative complication (odds ratio 0.38) was reported in recent meta-analysis concerning hemodynamic optimization [2]. Due to inclusion of intermediate risk patients we had expected to observe lower treatment effect. According to the rate of complications observed in the CONTROL group (33 patients (83 %)) and to treatment effect expected (conservative reduction from 83 to 50 %) a sample of 36 patients would be necessary (alpha and beta error of 0.05 and 0.2). We have decided to include 40 patients into each group in order to cover possible “drop outs” and also to facilitate the division into two strata (total hip vs. knee replacement).

The analysis was performed using the SigmaStat for Windows v.3.5 (Systat Software Inc., San Jose, USA). The Kolmogorov-Smirnoff test was used for normality assessment. For inter-group comparison one-way ANOVA or Kruskall-Wallis tests were used respectively. For time-dependent variables, repeated measures ANOVA or Friedman tests were performed. Post-hoc analysis was performed with the Student-Newman-Keul’s or Dunn’s test. Categorical variables were tested using the Chi-square test. The *p* < 0.05 was taken as statistically significant.

## Results

**Fig. 2:**
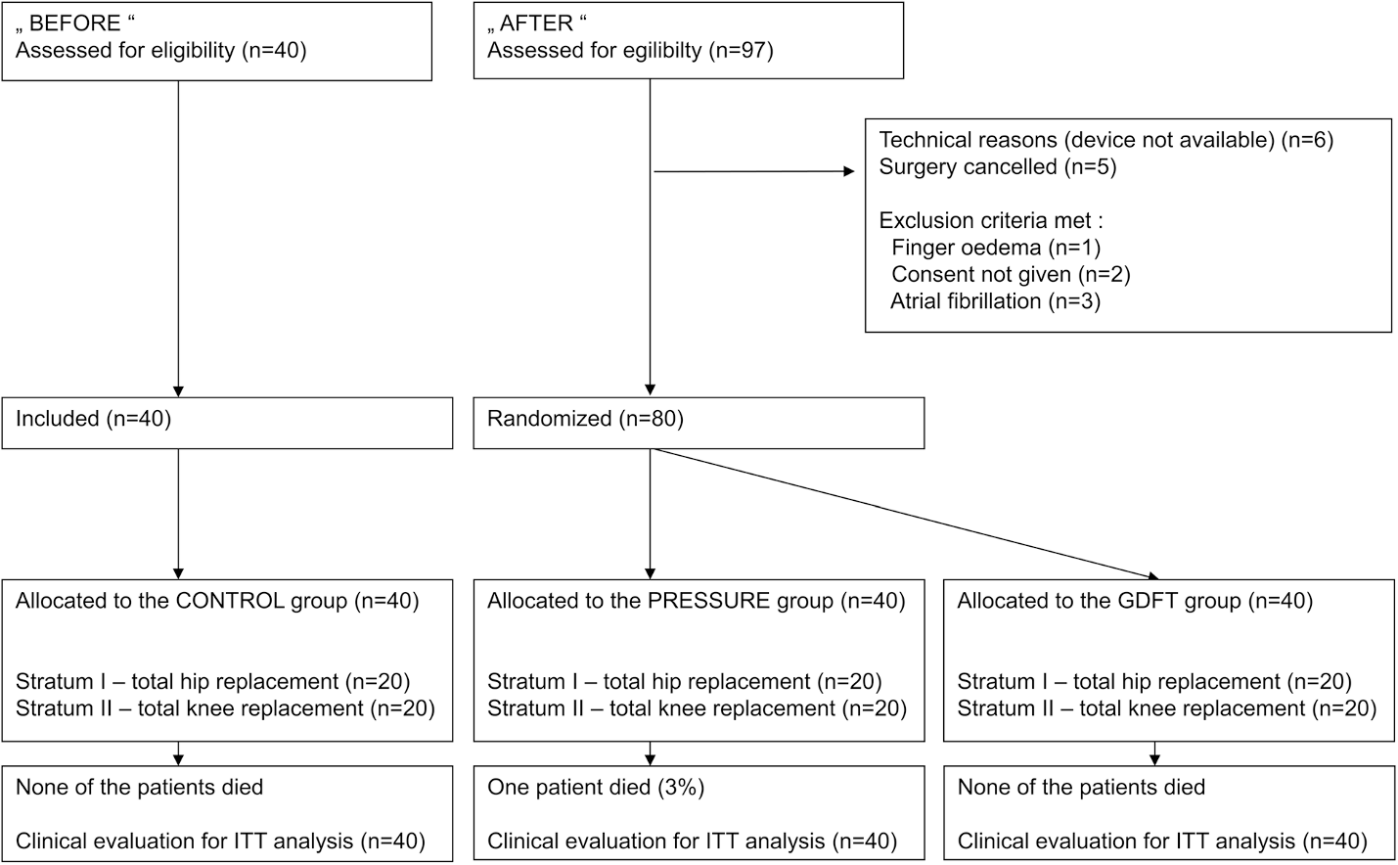
The flow chart of patients through the trial. Legend: ITT – intention-to-treat analysis

During the first stage (August and September 2012), 40 consecutive patients undergoing hip (20 patients) and knee (20 patients) replacements under general anesthesia were observed for peri- and postoperative outcomes (CONTROL group). All patients operated during this period were included and there were none lost to follow up. In the second stage (late November 2012 to early March 2013), a total of 97 hip or knee replacements were performed under general anesthesia and found eligible for study inclusion. Seventeen patients were excluded before randomization for various reasons (listed in Fig. 2), 80 patients were included and equally randomized into two groups each with two strata containing 20 patients. All patients in both stages gave informed consent and were included in the final analysis, one of them died within 30 postoperative days because of pulmonary embolization. The entire flow chart according to CONSORT statement is displayed in Fig. 2. No significant differences were observed between the three groups in regard to demographic parameters or chronic comorbidities (Table 1).

**Table 1:** Baseline and demographic characteristics

	CONTROL	PRESSURE	GDFT	*P* value
	(*N* = 40)	(*N* = 40)	(*N* = 40)	
Age (years)	70 (54–84)	66 (44–80)	68 (33–84)	0.145
Sex (F/M)	25/15	26 / 14	23/17	0.789
Height (cm)	167 (8)	166 (9)	169 (11)	0.385
Weight (kg)	82 (15)	82 (15)	90 (16) ^a^^b^	0.025
ASA (1/2/3)	2/23/15	7/24/9	6/27/7	0.164
Arterial hypertension	30 (75 %)	28 (70 %)	27 (68 %)	0.754
Ischemic heart disease	9 (23 %)	7 (18 %)	4 (10 %)	0.320
Chronic pulmonary disease	6 (15 %)	4 (10 %)	4 (10 %)	0.724
Diabetes mellitus	9 (23 %)	6 (15 %)	10 (25 %)	0.519

Data are presented as mean and range for age, mean (standard deviation) or number (proportion) for other parameters

^a^ - significant difference between PRESSURE vs. GDFT groups

^b^ - significant difference between protocolled group (GDFT or PRESSURE) vs. CONTROL

The results of primary and secondary outcome data are summarized in Tables 2 and 3. The rate of complications among patients of the CONTROL group was higher as compared to both protocol groups. In pair-wise comparisons, only the difference between CONTROL and GDFT reached statistical significance (33 patients (83 %) vs. 22 (55 %); *p* = 0.02; relative risk 0.67 (95 % confidence interval 0.49–0.91)). The higher number of complications resulted in a trend towards the prolongation of hospital length of stay as assessed by readiness for discharge criterion.

**Table 2:** Intervention data, fluid balance and laboratory outcome

	CONTROL	PRESSURE	GDFT	*P*-value
	(*N* = 40)	(*N* = 40)	(*N* = 40)	
Length of the procedure (min)	110 (100–120)	105 (90–118)	100 (85–113)	0.06
MAP before anesthesia (mmHg)	119 (15)	113 (17)	114 (19)	0.31
MAP end of procedure (mmHg)	118 (14)	105 (12)^b^^c^	103 (18)^b^^c^	<0.001
HR before anesthesia (beats per minute)	73 (10)	72 (11)	68 (13)	0.20
HR end of procedure (beats per minute)	74 (14)	78 (16)^c^	77 (14)^c^	0.34
PPV after induction	N/A	7.9 (4.2) %	8.9 (4.5) %	0.36
PPV end of procedure	N/A	7.2 (2.4) %	8.2 (3.8) %	0.15
Hypotensive periods intraoperatively	3 (1–5)	2 (1–4)	2 (0–4)	0.09
Ephedrin (number of interventions)	2 (0–3)	0 (0–1)^b^	0 (0–1)^b^	<0.001
Ephedrin (dose - mg)	10 (0–15)	0 (0–10)	0 (0–10)	0.11
Hemoglobin before anesthesia (g/l)	136 (11)	121 (12) ^a^^b^	131 (14)	<0.001
Hemoglobin end of procedure (g/l)	117 (15)	110 (13) ^a^^b^	118 (16)	0.03
Lactate end of procedure (mmol/l)	N/A	1.2 (0.4)	1.2 (0.6)	0.99
Intraoperative fluid balance				
Blood loss (ml)	500 (300–575)	500 (300–600)	400 (300–600)	0.77
Maintenance fluids (crystalloid) (ml)	1500 (1200–1500)	700 (600–750)^b^	750 (600–900)^b^	<0.001
Bolus fluids (colloid) (ml)	0 (0–500)	440 (100–500)^b^	400 (0–500)^b^	0.01
Patients receiving transfusion	5 (13 %)	3 (8 %)	1 (3 %)	0.24
Number of packed blood cells transfused	0 (0–0)	0 (0–0)	0 (0–0)	0.26
Early postoperative fluid balance (24 h)				
Blood loss (ml)	735 (400–805)	470 (390–840)	500 (380–678)	0.42
Diuresis (ml)	1300 (1025–1625)	1000 (785–1378)^b^	1075 (900–1300)^b^	0.01
Crystalloids (ml)	2525 (2300–2800)	2800 (2300–3150)	2700 (2350–3125)	0.27
Colloids (ml)	0 (0–0)	0 (0–0)	0 (0–0)	0.70
Patients receiving transfusion	17 (43 %)	11 (28 %)	4 (10 %)^b^	0.01
Number of packed blood cells transfused	0 (0–2)	0 (0–2)	0 (0–0)^b^	0.01
Hemoglobin (24 h) (g/l)	105 (10)	102 (12) ^a^	110 (14) ^a^	0.01
Lactate (24 h) (mmol/l)	N/A	1.6 (0.8)	1.7 (0.8)	0.67

Data are presented as mean (standard deviation), median (interquartil range) or number (proportion)

*PPV* pulse pressure variation, *HR* heart rate, *MAP* mean arterial pressure

^a^ - significant difference between PRESSURE vs. GDFT groups

^b^ - significant difference between protocolled group (GDFT or PRESSURE) vs. CONTROL

^c^ - significant difference against baseline

**Table 3:** Clinical outcome

	CONTROL	PRESSURE	GDFT	*P*-value
	(*N* = 40)	(*N* = 40)	(*N* = 40)	
Rate of complications (all)	33 (83 %)	26 (65 %)	22 (55 %)^a^	0.03
Rate of complications (major)	3 (8 %)	6 (15 %)	2 (5 %)	0.27
Mortality	0 (0 %)	1 (2.5 %)	0 (0 %)	0.37
Hospital length of stay (days)	10.5 (8–12)	10 (9–12.5)	10 (8.5–13.5)	0.99
Discharge readiness (days)	9 (8–12)	9 (7–12)	8 (7–10)	0.06
ICU length of stay (days)	2 (2–2)	2 (2–3)	2 (2–3)	0.3
ICU readiness (days)	2 (2–2)	2 (2–2)	2 (2–2)	0.38
Number of complications (all)	66	61	35	N/A
Number of complications (major)	3	8	3	N/A
Blood transfusion (patients)	30 (75 %)	25 (63 %)	15 (38 %)^a^	0.001
Blood transfusion (units)	2 (1–4)	2 (0–4)	0 (0–2)	N/A
Complications per group (Number of patients)				
Cardiovascular				
Minor	2 (5 %)	4 (10 %)	0 (0 %)	0.12
Major	0 (0 %)	1 (2.5 %)	1 (2.5 %)	0.6
Respiratory				
Minor	1 (2.5 %)	1 (2.5 %)	0 (0 %)	0.6
Major	0 (0 %)	1 (2.5 %)	0 (0 %)	0.37
Infectious				
Minor	23 (57.5 %)	9 (22.5 %)^a^	9 (22.5 %)^a^	0.001
Major	2 (5 %)	3 (7.5 %)	2 (5 %)	0.86
Renal				
Minor	5 (12.5 %)	7 (17.5 %)	5 (12.5 %)	0.76
Major	0 (0 %)	0 (0 %)	0 (0 %)	1
GIT				
Minor	20 (50 %)	17 (42.5 %)	11 (27.5 %)	0.12
Major	0 (0 %)	0 (0 %)	0 (0 %)	1
Nervous				
Minor	1 (2.5 %)	3 (7.5 %)	3 (7.5 %)	0.55
Major	0 (0 %)	0 (0 %)	0 (0 %)	1
Coagulation				
Minor	6 (15 %)	7 (17.5 %)	3 (7.5 %)	0.39
Major	1 (2.5 %)	2 (5 %)	0 (0 %)	0.36

Data are presented as median (interquartil range) or number (proportion)

*ICU* intensive care unit, *GIT* gastro-intestinal tract

^a^ - significant difference between protocolled group (GDFT or PRESSURE) vs. CONTROL

**Fig. 3:**
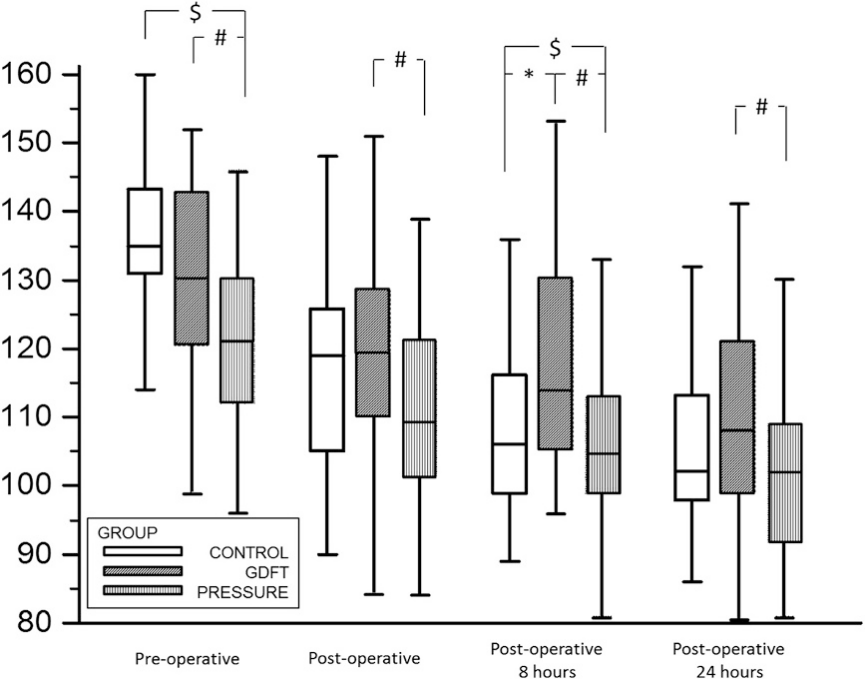
Haemoglobin levels in perioperative period. Legend: # - significant difference PRESSURE vs. GDFT groups; $ - significant difference PRESSURE vs. CONTROL groups; * - significant difference GDFT vs. CONTROL groups (all ANOVA with Student-Neuman-Keul’s post hoc analysis); the drop against baseline measurement was significant in all groups and time-points

In both protocol groups a trend for lower number of hypotensive periods was observed intraoperatively with lower dose of rescue vasoactive therapy. No significant differences were found between PPV values of both protocol groups (PRESSURE and GDFT). Pulse pressure variation was low in both groups in the beginning as well as at the end of the procedure. The overall fluid status (amount of fluids administered, blood loss etc.) was comparable in both protocol groups. Contrarily, the CONTROL patients received significantly higher amounts of fluid compared to both groups managed with protocol. A drop in hemoglobin level was observed among all patients, but was more pronounced in the CONTROL group patients (Fig. 3). A higher proportion of CONTROL patients needed transfusion during early as well as late postoperative periods. Serum lactate levels were assessed only in the prospective groups’ patients and showed no significant differences between PRESSURE and GDFT.

## Discussion

In our study, the adoption of a fluid administration protocol guided by pulse pressure variation assessed by continuous non-invasive arterial pressure monitoring seems to be associated with lower rate of postoperative complications compared to standard no-protocol care. Reduced amounts of infused fluids with maintained hemodynamic stability, lower dose of rescue vasoactive medication and lower rate of transfusion requirements were observed among patients of the GDFT group compared to CONTROL patients.

Fluid administration based on protocols with use of goal-directed hemodynamic optimization seems to be associated with improved postoperative outcomes in high risk surgical patients [3]. However, among intermediate risk patients, (for instance total hip or knee replacement) the evidence has been scarce and liberal strategies without proper monitoring are often accepted. Holte et al. [18] observed lower incidence in postoperative vomiting among patients managed with the liberal as opposed to the restrictive approach. The authors of this study hypothesized that preoperative fasting might influence this outcome and that a liberal regimen helps to cover the preoperative hypovolemia. According to values of PPV observed after induction, our patients were not hypovolemic before the procedure. In such cases a liberal approach might drive them into hypervolemia and resulting hemodilution. Red blood cell transfusions can exhibit immunosuppressive effect [19] and lead to increased incidence of infectious complications. In our study the protocol approach to fluid administration itself (PRESSURE group) was associated with a trend towards better clinical outcomes and lower transfusion needs. But it seems that only a complex protocol (restrictive maintenance with fluid responsiveness assessment- GDFT group) can adapt for the variable blood loss and individual needs. This is supported by our results or by the study by Cecconi [13]. In that trial, goal-directed fluid management was further combined with dopexamine infusion in order to reach a predefined goal of oxygen delivery. Lower incidence of cardiac and minor organ complications was observed, resembling our data. However, the use of inotropic support might be questioned in patients without significant cardiac morbidity and the use of arterial catheters is also not standard for these procedures.

The outcomes of the GDFT group patients show that fluid optimization itself might be sufficient in this intermediate risk population. Recent meta-analysis demonstrated that dynamic predictors of fluid responsiveness are useful as goals of perioperative GDT [11]. Using them is much easier than complex cardiac output monitoring and oxygen delivery calculation. In addition, the number of limitations in intermediate risk groups seems to be much lower than reported by Maguire [12]: atrial fibrillation was present only in 3 (3 %) out of 97 eligible patients and no other limitations for the use of PPV were observed among our patients. The ventilation strategy (tidal volume of 8 ml/kg and positive end expiratory pressure of 0.6 kPa) is in line with recent recommendations for intraoperative protective ventilation [20], but still enabling the use of dynamic variations. An important aspect of our trial is that the protocol was based on measurements obtained totally non-invasively.

Our study is the first one using the novel non-invasive continuous pressure device based on volume-clamped method for goal-directed treatment. Both the reliability and limits of the measurement should be recognized, even though the device is marketed worldwide and approved by most certification authorities. As for now three large scale [21–23] and multiple smaller studies demonstrated the validity of the pressure values obtained by the CNAP® monitor, showing acceptable agreement with direct arterial pressure monitoring. Some inaccuracies seem to exist in cases of profound pressure fluctuations (i.e. deep hypotension following anesthesia induction etc.). The trending ability remained unaffected by these inaccuracies [24, 25]. In addition, as pointed out by Hahn [22], the device uses the oscillometric cuff as a reference, making it equivalent to contemporary practice in the studied population. It was demonstrated by our group [26] as well as by others [27, 28] that the use of continuous non-invasive pressure monitoring devices enables faster recognition of blood pressure drops and helps to maintain the hemodynamic stability during surgery. The reliability of the PPV values obtained by the CNAP® monitor was tested recently by two studies and was found to be comparable to its invasively assessing counterparts [29, 30]. Given these positive factors, the CNAP® device is already widely and routinely used in many clinical institutions; therefore our study could serve as a proof of concept for this praxis.

The design of our study poses an important selection and assessment bias and hence limits the generalizability of our results. Firstly, the use of a two-stage (“before and after”) design does not allow us to compare the CONTROL group and protocol groups in parallel. However, our aim was to quantify the effect of change in approach during the unique moment of transition between no-protocol and protocol-led care. In order to avoid selection bias, all patients undergoing the procedure within the defined stages were found eligible. In the first stage there were no dropouts and only a limited number of patients was excluded during the second stage. Secondly, our study was not set to evaluate between the groups of phase two so we can derive only indirect conclusions in regard of superiority of the goal-directed fluid optimization over the restrictive regimen. This observed difference resulted mainly from the lower incidence of minor infectious complications (mostly urinary tract and surgical site infections). Besides blood and fluids management this might also be confounded by a slightly uneven distribution of comorbidities. Even though not statistically significant, patients in the GDFT group tended to be healthier. This could influence both the transfusion trigger and also the risk for complications as well as help reach the predefined readiness for discharge criteria earlier.

Beside these specific limitations, our trial suffers from flaws inherent to single centre studies with “keen investigators” and a low number of patients. This might be especially true for intermediate risk patients with a low number of complications. For this reason our conclusions should be regarded as a hypothesis generating at best. A much larger study would be necessary to prove this concept. However, it seems that the use of the continuous non-invasive arterial pressure device might offer a safe alternative for monitoring and enable better adoption of fluid protocols.

## Conclusions

In conclusion, in our study the transition from standard no-protocol treatment to the fluid management protocol based on pulse pressure variation assessed by continuous non-invasive arterial pressure measurement seems to be associated with reduction of postoperative infections, of organ complications, and of transfusion needs.

## Additional files

Additional file 1: **The CONSORT Check list.** (DOC 217 kb) Additional file 2: **List of predefined morbidity criteria for ICU and hospital discharge and list of complications.** (DOCX 15 kb)

## Abbreviations

ASA: American Society of Anesthesiology physical status
CNAP: Continuous Non-invasive Arterial Pressure (name of the device)
CONTROL: Name of the prospective observational group
GDFT: Name of the randomized group with fluid management based on pulse pressure variation
GDT: Goal-directed therapy
GIT: Gastro-intestinal tract
HR: Heart rate
ICU: Intensive Care Unit
MAC: Minimal alveolar concentration
MAP: Mean arterial pressure
PPV: Pulse pressure variation
PONV: Postoperative nausea and vomiting
PRESSURE: Name of the randomized group based on standard fluid management
WHO: World Health Organization
