# Supplementary material for: Fluid management guided by a continuous non-invasive arterial pressure device is associated with decreased postoperative morbidity after total knee and hip replacement
Source: BMC Anesthesiol. 2015 Oct 15;15:148. doi: 10.1186/s12871-015-0131-8 (PMC4608292; doi:10.1186/s12871-015-0131-8)
Supplement: Additional file 2: — List of predefined morbidity criteria for ICU and hospital discharge and list of complications. (DOCX 15 kb) [file 12871_2015_131_MOESM2_ESM.docx]

| **Electronic supplementary material:**  **Table 1 – Discharge readiness criteria and complications definition** | | |
| --- | --- | --- |
| ICU Readiness for discharge | Haemodynamically stable without intravenous vasoactive medication  Without marked dyspnoea and not dependent on oxygen  Without signs of confusion or delirium  Without signs of major complication or severe organ dysfunction | |
| Hospital readiness for discharge | No signs of organ dysfunction (cardiovascular, respiratory, GIT etc.)  Analgesia sufficient on per-oral medication  No overt signs of infection (or stable without need for i.v. antimicrobial treatment)  Satisfactory return of pre-morbid mobility | |
|  | | |
| **Complications** | Minor | Major |
| Cardiovascular | - Severe hypotension or hypertension requiring pharmaceutical intervention - Non-severe arrhythmia | - Acute myocardial infarction - Heart failure - Severe arrhythmia |
| Respiratory | - Mild dyspnoea requiring oxygenotherapy | - Severe dyspnoea requiring invasive/non/invasive ventilator support |
| Infectious | - Localised surgical site infection - Urinary tract infection including asymptomatic bacteriuria - Other non-severe infect requiring antimicrobial treatment | - Surgical site infection requiring reoperation or drainage - Pneumonia - Sepsis |
| Renal | - AKIN 1^st^ stage | - AKIN 2^nd^ and 3^rd^ stage |
| GIT | - Nausea and vomiting - Diarrhoea - Paralytic ileus | - GIT bleeding - Liver failure |
| Nervous system | - Cognitive dysfunction/delirium   requiring medication | - Stroke |
| Coagulation | - Local bleeding - Deep vein thrombosis | - Bleeding requiring surgical re-intervention - Pulmonary embolism |
